# Dissecting the Role of Critical Residues and Substrate Preference of a Fatty Acyl-CoA Synthetase (FadD13) of *Mycobacterium tuberculosis*

**DOI:** 10.1371/journal.pone.0008387

**Published:** 2009-12-21

**Authors:** Garima Khare, Vibha Gupta, Rakesh K. Gupta, Radhika Gupta, Rajiv Bhat, Anil K. Tyagi

**Affiliations:** 1 Department of Biochemistry, University of Delhi South Campus, New Delhi, India; 2 Department of Microbiology, Ram Lal Anand College, University of Delhi South Campus, New Delhi, India; 3 School of Biotechnology, Jawaharlal Nehru University, New Delhi, India; Universita di Sassari, Italy

## Abstract

Newly emerging multi-drug resistant strains of *Mycobacterium tuberculosis* (*M.tb*) severely limit the treatment options for tuberculosis (TB); hence, new antitubercular drugs are urgently needed. The *mymA* operon is essential for the virulence and intracellular survival of *M.tb* and thus represents an attractive target for the development of new antitubercular drugs. This study is focused on the structure-function relationship of Fatty Acyl-CoA Synthetase (FadD13, Rv3089) belonging to the *mymA* operon. Eight site-directed mutants of FadD13 were designed, constructed and analyzed for the structural-functional integrity of the enzyme. The study revealed that mutation of Lys^487^ resulted in ∼95% loss of the activity thus demonstrating its crucial requirement for the enzymatic activity. Comparison of the kinetic parameters showed the residues Lys^172^ and Ala^302^ to be involved in the binding of ATP and Ser^404^ in the binding of CoenzymeA. The influence of mutations of the residues Val^209^ and Trp^377^ emphasized their importance in maintaining the structural integrity of FadD13. Besides, we show a synergistic influence of fatty acid and ATP binding on the conformation and rigidity of FadD13. FadD13 represents the first Fatty Acyl-CoA Synthetase to display biphasic kinetics for fatty acids. FadD13 exhibits a distinct preference for C_26_/C_24_ fatty acids, which in the light of earlier reported observations further substantiates the role of the *mymA* operon in remodeling the cell envelope of intracellular *M.tb* under acidic conditions. A three-dimensional model of FadD13 was generated; the docking of ATP to the active site verified its interaction with Lys^172^, Ala^302^ and Lys^487^ and corresponded well with the results of the mutational studies. Our study provides a significant understanding of the FadD13 protein including the identification of residues important for its activity as well as in the maintenance of structural integrity. We believe that the findings of this study will provide valuable inputs in the development of inhibitors against the *mymA* operon, an important target for the development of antitubercular drugs.

## Introduction

*Mycobacterium tuberculosis (M.tb)*, an intracellular pathogen, is exquisitely adapted for human parasitization [1]. It has evolved a number of distinct strategies to survive in the hostile environment of macrophages [2]. The drugs for the treatment of tuberculosis (TB) are available but the long and demanding regimens lead to erratic and incomplete treatment often resulting in the development of drug resistance. Hence, the importance of identification and characterization of new drug targets cannot be overemphasized.

*M.tb* has a unique and large repertoire of lipid associated genes [3] and its cell wall, which is known to contain a distinct variety of lipids, plays a crucial role in its pathogenesis [4]. The pathogen resides in the host macrophages, where it encounters various stressful conditions such as changes in pH, exposure to reactive oxygen, nitrogen intermediates, degradative enzymes and deprivation of essential nutrients [5]. During these conditions, the lipid rich cell surface of *M.tb* is often subjected to damage by the host assault. Hence, this pathogen has developed a variety of means to modify its cell envelope [6] for its survival in the hostile environment, emphasizing the importance of its cell envelope constituents as targets for the development of new antitubercular drugs. It has been earlier demonstrated that exposure to acidic pH results in the upregulation of the *mymA* operon of *M.tb* (Rv3083 - Rv3089) [7], [8]. The functional loss of the *mymA* operon leads to alterations in the colony morphology, cell wall structure, mycolic acid composition and drug sensitivity and results in markedly reduced intracellular survival of *M.tb* in macrophages [8], [9], [10]. Besides, the *mymA* mutant of *M.tb* shows a drastic reduction (800 fold) in its ability to survive in the spleen of guinea pigs as compared to the parental strain [9]. To gain further insight into the functioning of *mymA* operon, a potential target for developing antitubercular drugs, it is necessary to characterize its gene products.

*fadD13*, the last gene of the *mymA* operon, encodes a Fatty Acyl-CoA Synthetase. Fatty Acyl-CoA Synthetases are ubiquitously distributed from bacteria to mammalian systems [11] and catalyze the activation of various fatty acids by converting them into fatty acyl-CoA thioesters [12]; the latter are shown to be important for the synthesis of triacylglycerols and phospholipids [13]. Moreover, many Acyl-CoA Synthetases have been suggested and identified as potential drug targets in the cases of several human pathogens [14], [15], [16]. Mechanistically, these proteins carry out the catalysis in two steps involving fatty acids, ATP and CoA [12]. In the first step, the fatty acid and the ATP react to form the fatty acyl-AMP intermediate with the release of pyrophosphate. The fatty acyl group is then transferred to the thiol group of the CoA acceptor to form fatty acyl-CoA with the concomitant release of AMP [12]. The reaction mechanism followed by this class of enzymes is:
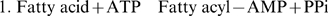


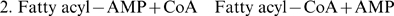



This study is focused on the characterization and structure-function relationship of Fatty Acyl-CoA Synthetase (Rv3089, FadD13) of the *mymA* operon and describes the influence of several mutations on the activity and structural integrity of the enzyme thus leading to the identification of residues important for its function. The relationship of the experimentally determined important residues with the structural arrangement of the catalytic centre is validated by homology modeling of FadD13. Based on the comparative assessment of fatty acids as substrates, FadD13 belongs to the class of Very Long chain Fatty Acyl-CoA Synthetases.

## Results

### Purification and Analysis of Aggregation Properties of the Recombinant FadD13

**Figure 1:**
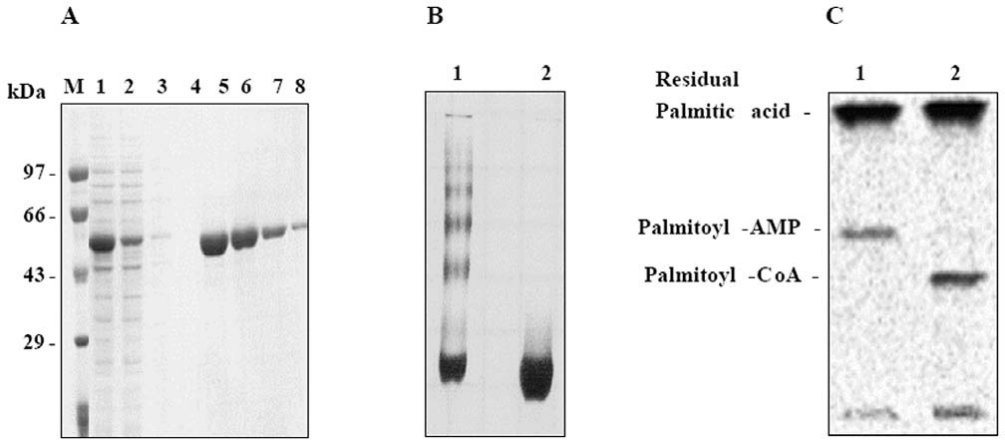
Purification, aggregation property and activity of FadD13 of *M. tuberculosis*. A. Analysis of purified FadD13. The purification was carried out by using Ni-NTA affinity chromatography and analyzed by electrophoresis on a 10% SDS-polyacrylamide gel. M – Molecular weight markers, lane 1- cell free extract, lane 2- unbound proteins, lane 3 – wash with lysis buffer containing 20 mM imidazole, lane 4 – wash with lysis buffer containing 50 mM imidazole, lane 5–8 – elutions with lysis buffer containing 250 mM imidazole. B. Analysis of aggregation property. The aggregating nature of FadD13 was studied by using 7.5% non-reducing non-denaturing polyacrylamide gel. Lane 1–20 µg of the native protein, lane 2–20 µg of the native protein incubated with 10 mM DTT and 1 mM ATP for 2 hours. C. Determination of the enzymatic activity of FadD13. FadD13 assay was carried out by using a radioactivity based TLC assay as described in the “Materials and Methods”. Figure shows the reaction products in the absence (lane 1) and presence (lane 2) of CoenzymeA.

*fadD13* gene was cloned in pET28c and expression and localization of the gene product was analyzed by SDS-electrophoresis using a 10% polyacrylamide gel. A significant expression of *fadD13* gene product as a protein of an apparent molecular weight of ∼57 kDa (comprising of the native protein of 55 kDa plus 2 kDa contributed by the histidine tag and the linker amino acids) was observed with the majority of the recombinant protein in the soluble fraction (data not shown). The N-terminal 6x histidine tagged recombinant protein was purified by using Ni-NTA affinity chromatography. The purified FadD13, as seen in Fig. 1A, was found to be highly pure (lanes 5–8). The native gel electrophoresis, as shown in Fig. 1B (lane 1), strongly suggested that FadD13 has a tendency to aggregate. The observed aggregation was marginally reduced in the presence of 1 mM ATP or 10 mM DTT, though to a greater extent in the case of DTT. However, the addition of 1 mM ATP and 10 mM DTT together completely abolished the aggregation, as shown in Fig. 1B (lane 2), suggesting that disulfide crosslinking acts as one of the major determinants in the formation of these aggregates. It was also noted that this aggregation was largely concentration dependent as dilution of the protein relieved the aggregation. However, the activity of FadD13 remained uninfluenced by this aggregation as both the forms of FadD13 (aggregated v/s disaggregated form) exhibited comparable activities (data not shown).

### Demonstration of Fatty Acyl-CoA Synthetase Activity of FadD13

**Figure 2:**
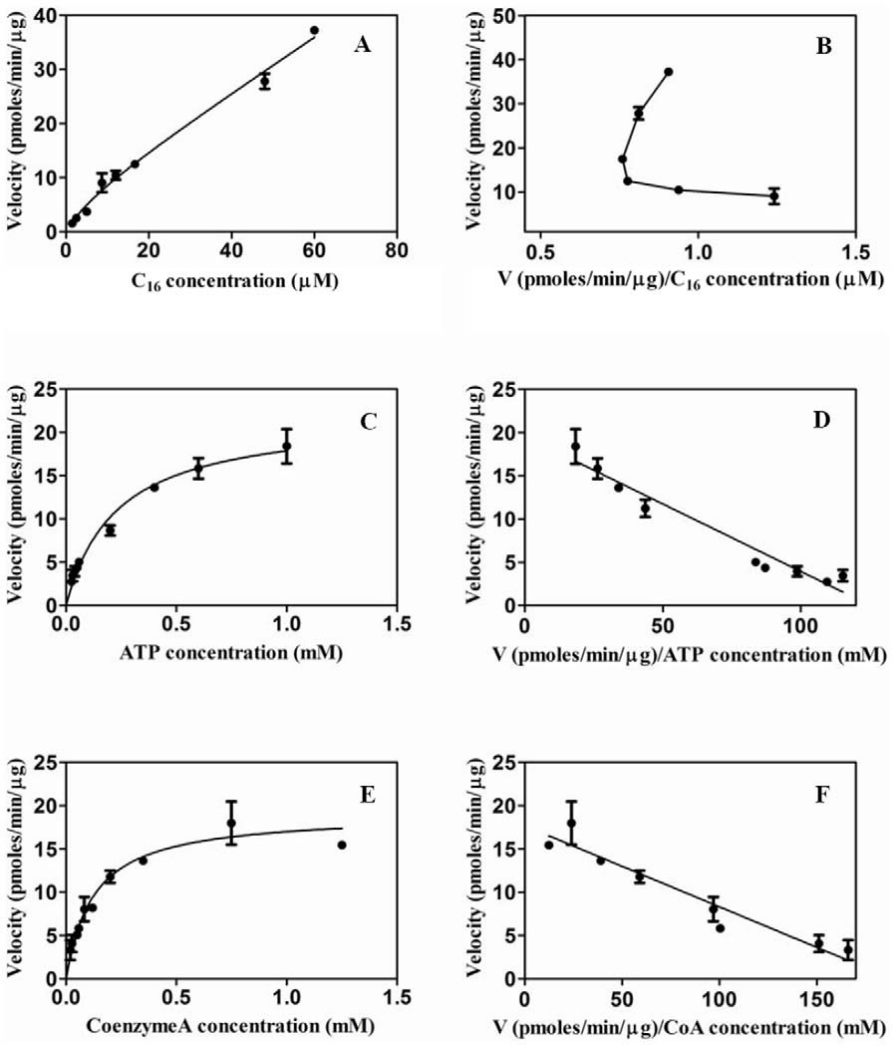
Analysis of kinetic behavior of FadD13 for various substrates. FadD13 assays were performed by varying the concentrations of a particular substrate under the standard conditions of the assay as described in the section on “Materials and Methods” and the kinetic behavior was analyzed by plotting Michaelis-Menten curve as well as Eadie-Hofstee plot. Michaelis-Menten curve (A) and Eadie-Hofstee plot (B) by using palmitic acid as the variable substrate, Michaelis-Menten curve (C) and Eadie-Hofstee plot (D) by using ATP as the variable substrate, Michaelis-Menten curve (E) and Eadie-Hofstee plot (F) by using CoenzymeA as the variable substrate. The data is depicted as mean of values ± S.E. of two experiments carried out in duplicates.

The Fatty Acyl-CoA Synthetase activity was determined by using radiolabeled palmitic acid(s) as described in “Materials and Methods”. As depicted in Fig. 1C (lane 2), FadD13 converted palmitic acid to palmitoyl-CoA in the presence of ATP and CoA. The presence of the reaction intermediate namely palmitoyl–AMP is also clearly shown in lane 1 (Fig. 1C), which appeared as a result of withholding CoA from the reaction mixture. The residual fatty acid, fatty acyl-AMP and fatty acyl-CoA migrate on the TLC with the Rf values of 0.95, 0.56 and 0.43, respectively, which are in accordance with the Rf values documented for these compounds previously [17].The V_max_ and K_cat_ values of the enzyme were estimated to be 14.62±0.30 pmoles/min/µg and 0.03 sec^−1^, respectively. FadD13 displayed a classical Michaelis-Menten kinetics for its co-factors ATP and CoA (Fig. 2-C,D,E and F) with K_m_ values of 0.23±0.05 mM and 0.13±0.002 mM, respectively. However, fatty acids, the substrate for the enzyme activity showed a biphasic kinetics with a K_m_ value of 19.79±5 µM (Fig. 2A and 2B, data shown for palmitic acid).

### Preference of FadD13 for Very Long Chain Fatty Acids

**Figure 3:**
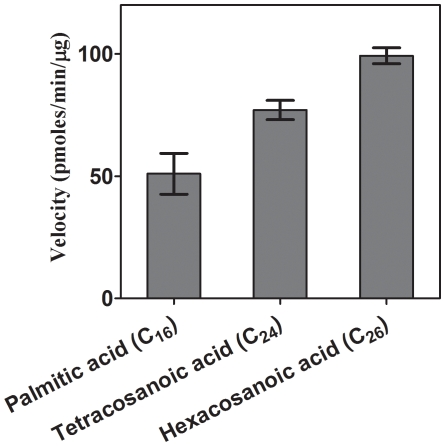
Evaluation of the preferance of FadD13 for fatty acids as substrate. The C_24_/C_26_ fatty acids were solubilized in α-cyclodextrin and the activity of FadD13 was measured as described in “Materials and Methods”. The final concentration of a fatty acid and α-cyclodextrin in the assay mixture was 15 µM and 4 mM. The data is depicted as mean of values ± S.E. of three independent experiments.

To ascertain the preference of FadD13, fatty acids of various chain lengths viz palmitic acid (C_16_), tetracosanoic acid (C_24_) and hexacosanoic acid (C_26_) were separately employed as a substrate for FadD13. The higher chain length fatty acids (C_24_ and C_26_) were solubilized in α-cyclodextrin as described in earlier studies [18], [19] and the reactions were carried out in the presence of 15 µM fatty acids and 4 mM α-cyclodextrin. The enzyme exhibited appreciable activity with all the three substrates. However, as is evident from Fig. 3, FadD13 exhibited the highest activity with C_26_ fatty acid followed by C_24_ fatty acid. Palmitic acid (C_16_) exhibited the least activity amongst the three. Thus, FadD13 exhibited preference for the fatty acid chain length in a rank order of C_26_>C_24_>C_16_ with an activity ratio of 100∶77∶50. Hence, FadD13 belongs to the class of Very long chain Fatty Acyl-CoA Synthetases (VLFACS).

### Molecular Assembly of FadD13

**Figure 4:**
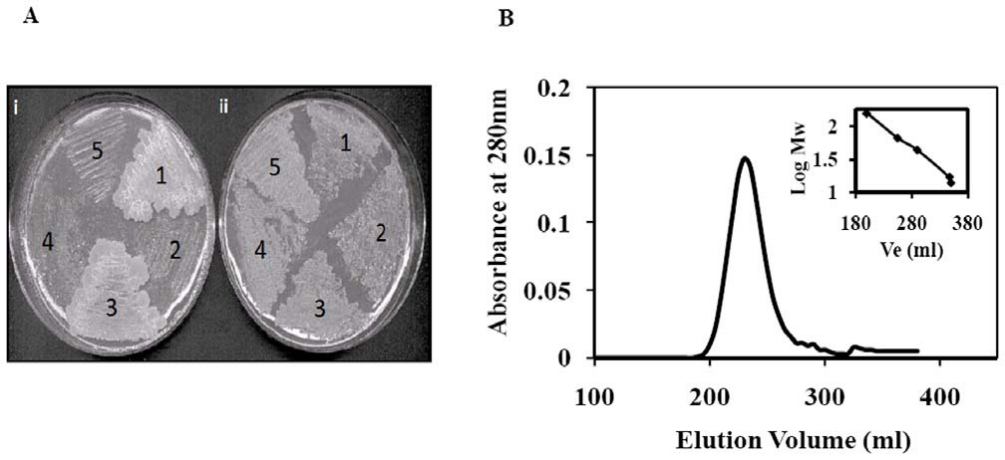
Determination of molecular assembly of FadD13. A. Determination of oligomerisation status of FadD13 by using M-PFC method. *M. sm* cells were independently electroporated with M-PFC plasmids producing following: (1) – FadD13_[F1,2]_/FadD13_[F3]_, (2)–*hsp60*_[F1,2]_/*hsp60*_[F3]_, (3)– GCN4_[F1,2]_/GCN4_[F3]_, (4) – FadD13_[F1,2]_/*hsp60*_[F3]_, (5) – *hsp60*_[F1,2]_/FadD13_[F3]_. Transformants obtained on 7H11 Agar Kan/Hyg plates were again streaked on (i) - 7H11 Agar Kan/Hyg/Trim and (ii) – 7H11 Agar Kan/Hyg plates, which were incubated at 37°C for ∼7 days before scoring. B. Determination of subunit composition of purified FadD13. Gel-filtration chromatography was performed by using a Sephadex G-200 column. Figure shows the elution profile of FadD13. The inset shows the calibration curve for the calculation of the molecular weight of unknown protein prepared by using the Biorad standard markers (Ve – elution volume).

For determining the oligomeric status of FadD13, we employed Mycobacterial-Protein Fragment Complementation (M-PFC) method [20], which is based upon the functional reconstitution of two small murine dihydrofolate reductase domains independently fused to two proteins, whose interaction has to be determined. The gene encoding FadD13 was cloned in the vectors pUAB300 and pUAB400, leading to the production of FadD13_[F1,2]_ and FadD13_[F3]_, respectively. The *M.sm* cells were co-electroporated with the plasmids and the transformants obtained were streaked on trimethoprim. All the appropriate controls were included in the experiment. Growth was observed in the case of positive control [20] GCN4_[F1,2]_/GCN4_[F3]_ and FadD13_[F1,2]_/FadD13_[F3]_ as shown in Fig. 4A suggesting that FadD13 has an oligomeric assembly in the cellular milieu. Importantly, no growth was observed in the case of negative controls such as *hsp60*_F_[_1,2_]/*hsp60*_F_[_3_], FadD13_F_[_1,2_]/_F_[_3_], and _F_[_1,2]_/FadD13_[F3]_.

Subunit assembly of purified FadD13 was determined by gel-permeation chromatography by using a sephadex G-200 column. The protein eluted as a single peak (Fig. 4B) with a retention volume corresponding to a dimeric assembly (∼114 kDa).

### Engineering FadD13 to Identify Functionally and Structurally Critical Residues

**Figure 5:**
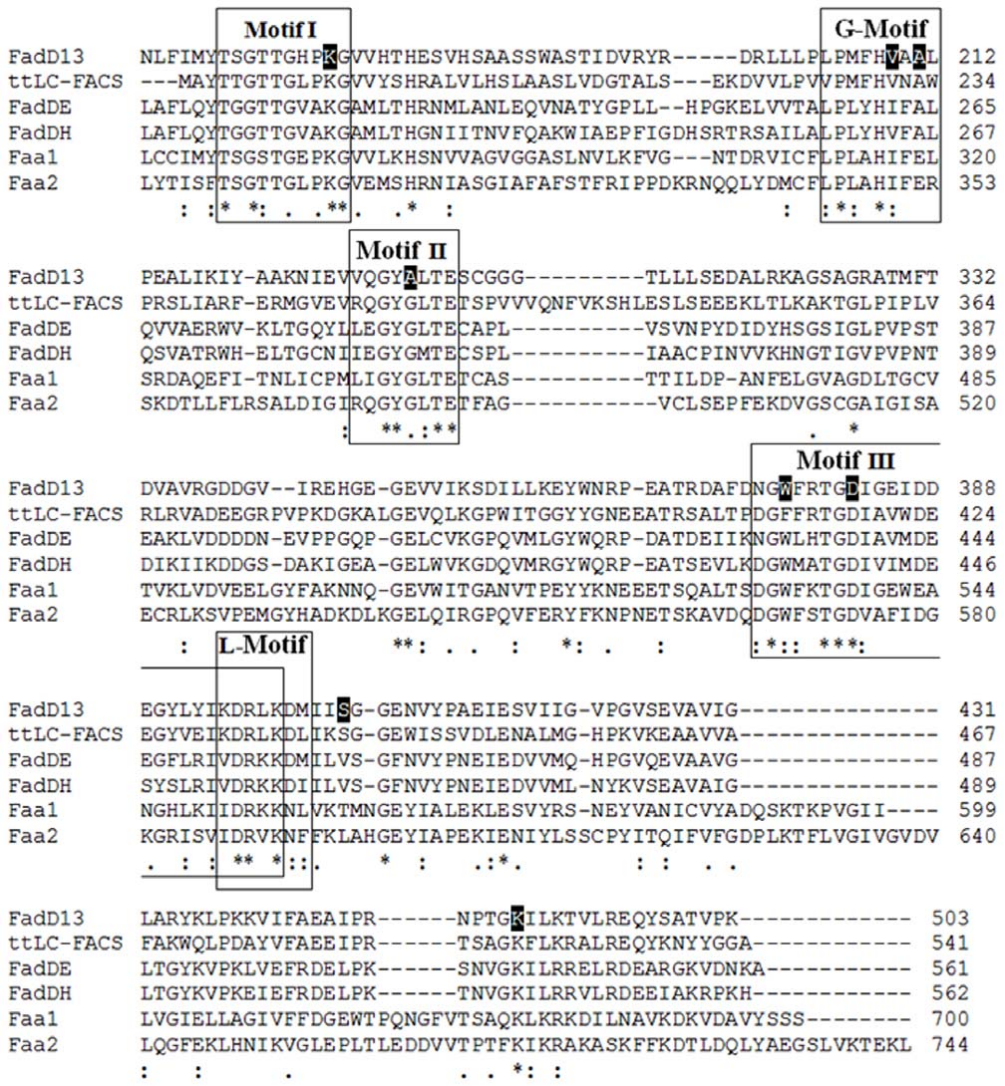
Comparison of FadD13 with the proteins of Fatty Acyl-CoA Synthetase family. The multiple sequence alignment was generated by using the ClustalW software. FadD13 – Fatty Acyl-CoA Synthetase from *M. tuberculosis*, ttLC-FACS - long chain-Fatty Acyl CoA Synthetase from *Thermus thermophilus*, FadDE – Fatty Acyl-CoA Synthetase from *Escherichia coli*, FadDH - Fatty Acyl-CoA Synthetase from *Haemophilus influenzae*, Faa1- Fatty Acyl-CoA Synthetase 1 from *Saccharomyces cerevisiae*, Faa2 - Fatty Acyl-CoA Synthetase 2 from *Saccharomyces cerevisiae*. Asterisks and dots indicate identical and similar residues, respectively. Double dots indicate higher similarity. Boxed residues comprise the phosphate-binding loop (Motif I), the gate motif (G-Motif), adenine binding motif (Motif II), fatty acid binding region (Motif III) and the linker region (L-Motif). Shaded residues depict the amino acids selected for mutagenesis.

A detailed comparison of FadD13 with the proteins of Fatty Acyl-CoA Synthetase family identified three major conserved regions (Fig. 5). The most highly conserved motifs I and II of FadD13 form the ATP/AMP binding site [21]. Of these, motif I (^164^TSGTTGHPKG^173^) corresponds to the P-loop proposed to bind to the phosphate group of ATP [21] whereas another conserved motif II (^298^VQGYALTE^305^) is similar to the A-motif involved in the binding to the adenine group of ATP/AMP in all known Fatty Acyl-CoA Synthetases [21]. A stretch of ∼25 amino acids (^375^NGWFRTGDIGEIDDEGYLYIKDRLKDM^401^), the III^rd^ highly conserved motif shows similarity to the signature motif, which is common to all Fatty Acyl-CoA Synthetases and is known to comprise the fatty acid binding site [22]. Additionally, a linker motif (L-motif) comprising of 7 amino acids (^395^KDRLKDM^401^) corresponds to the region that connects the C and N terminal domains of the protein in all homologs [23]. ^204^LPMFHVAAL^212^ residues show homology to the 9-residue gate motif (G-motif), which in homologous proteins interact with the residues of the C- terminal domain after the AMP moiety is bound to the protein and thus keeps the enzyme in the closed conformation required for catalysis [23]. This motif is also involved in interaction with the hydrophobic part of the fatty acyl chain [23]. Based on the conserved motifs, eight important residues were identified for the mutational studies of FadD13. These mutations were: K172A, V209D, A211G, A302G, W377A, D382A, S404A and K487A. The mutagenic oligonucleotides used for generating site-directed mutants of FadD13 are listed in Table 1. Lys^172^ and Ala^302^ belong to the ATP/AMP binding motifs I and II, respectively. The residues homologous to Lys^172^ are proposed to bind to the phosphate group of ATP via electrostatic interactions. Ala^302^ is a unique substitution in mycobacterial FadDs, which in most other cases is represented by a glycine residue known to bind the adenine group of ATP/AMP via hydrophobic interactions. Residues Val^209^ and Ala^211^ are a part of the gate motif and are also thought to be involved in the interaction with the hydrophobic part of the fatty acids. Residues Asp^382^ and Trp^377^ lie in the region III containing the signature motif of Fatty Acyl-CoA Synthetases involved in binding of fatty acids and guiding the preference of these proteins for fatty acids of various chain lengths [22]. Lys^487^ is a highly conserved residue; the corresponding residue in other homologs is involved in determining the orientation of the substrates thus leading to the formation of adenylate intermediate [24], [25]. Residue Ser^404^ present in the C-terminal domain of FadD13 was targeted, as serine residues from active site of several CoA utilizing enzymes are known to interact with 4′-phopshopantetheinyl moiety of CoA through their hydroxyl groups [26], [27], [28]. There was no significant change in the total expression levels (including cytoplasmic and inclusion bodies) of these mutants as compared to the native protein, however, the cytoplasmic expression was marginally reduced in the cases of mutants V209D and D382A and a markedly low cytosolic expression was observed in the case of mutant W377A (Fig. S1). To obtain the mutant W377A in the cytosolic fraction, the induction was carried out at 18°C for 16 hours.

**Table 1:** Mutagenic oligonucleotides used for generating site-directed mutants of FadD13.

Name	Oligonucleotide sequence*
K172A (F)	5′ CACCACCGGACATCCCGCGGGAGTGGTGCATACC 3′
K172A (R)	5′ GGTATGCACCACTCCCGCGGGATGTCCGGTGGTG 3′
V209D (F)	5′ CTGCCGATGTTCCACGACGCGGCGTTGACGACG 3′
V209D (R)	5′ CGTCGTCAACGCCGCGTCGTGGAACATCGGCAG 3′
A211G (F)	5′ CCGATGTTCCACGTGGCGGGGTTGACGACGGTCATC 3′
A211G (R)	5′ GATGACCGTCGTCAACCCCGCCACGTGGAACATCGG 3′
A302G (F)	5′ GGTCGTGCAGGGTTACGGACTCACCGAATCCTGTGGC 3′
A302G (R)	5′ GCCACAGGATTCGGTGAGTCCGTAACCCTGCACGACC 3′
W377A (F)	5′ GCTTTCGACAACGGTGCGTTCCGGACCGGCGAC 3′
W377A (R)	5′ GTCGCCGGTCCGGAACGCACCGTTGTCGAAAGC 3′
D382A (F)	5′ GTTCCGGACCGGCGCCATCGGCGAAATCGATG 3′
D382A (R)	5′ CATCGATTTCGCCGATGGCGCCGGTCCGGAAC 3′
S404A (F)	5′ GAAGGACATGATCATTGCCGGCGGCGAGAACGTG 3′
S404A (R)	5′ GACGTTCTCGCCGCCGGCAATGATCATGTCCTTC 3′
K487A (F)	5′ CCCCGCAACCCGACCGGCGCGATCCTCAAAACGGTG 3′
K487A (R)	5′ CACCGTTTTGAGGATCGCGCCGGTCGGGTTGCGGGG 3′

*mutagenic regions in the sequence are underlined.

### Effect of Site Directed Mutations on the Activity of FadD13 and Binding Affinity of the Cofactors

**Figure 6:**
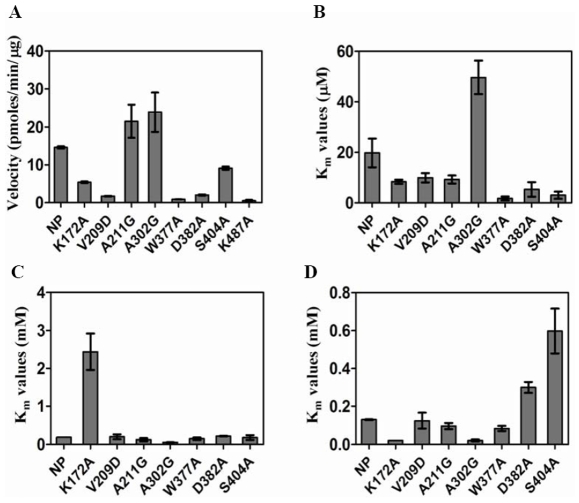
Comparison of the kinetic properties of FadD13 and its mutants. K_m_ values for substrates and V_max_ values were determined by performing FadD13 assays as described in the section on “Materials and Methods”. For the calculation of Michaelis constants, a varying range of substrate concentrations (Palmitic acid – 1.5–60 µM, ATP - 0.03–4 mM, CoenzymeA - 0.02–1.5 mM) were employed and the K_m_ values were calculated by GraphPad Prism 5 (San Diego, California, USA) by using a non-linear fitting method. (A) Comparison of V_max_ values of the native FadD13 (NP) and its mutants. K_m_ values of Palmitic acid (B), ATP (C) and CoenzymeA (D) for native FadD13 (NP) and its mutants. The data is depicted as mean of values ± S.E. of two independent experiments carried out in duplicates.

The mutations of the targeted residues influenced the catalytic activity of the protein suggesting their involvement in the mechanism of enzymatic action (Fig. 6A). The most drastic influence on the enzyme activity was demonstrated by the mutants D382A, V209D, W377A and K487A resulting in a sharply declined activity (a reduction by 85.9%, 87.9%, 93.9% and 96.3% in the activity and 7.1 fold, 8.39 fold, 17.3 fold and 27.7 fold in the K_cat_ values, respectively) as compared to the activity of the native protein. Mutations of the residues Ser^404^ and Lys^172^ also resulted in the reduced activity of FadD13; however, the magnitude of reduction in these cases was not as drastic, with a reduction of 39.2% and 63.5% in the activity as well as 1.6 fold and 2.7 fold decrease in the K_cat_ values, respectively in comparison to the native protein. The Mutants A211G and A302G did not result in any loss of the enzyme activity, infact, the enzymatic activity as a result of these mutations increased by 20% and 66.7%, respectively, with an approximate 1.5 fold enhanced K_cat_ values. Comparative enzymatic activities of the native protein and the mutants are summarized in Table 2.

**Table 2:** Comparison of the kinetic parameters of native FadD13 and its mutants*.

Protein	V_max_ (pmoles/min/µg)	k_cat_ (sec^−1^)	K_m_ for Palmitic acid (µM)	K_m_ for ATP (mM)	K_m_ for CoA (mM)
FadD13	14.62±0.30	.0277	19.79±5.67	0.24±0.05	0.13±0.01
K172A	05.41±0.20	.0102	08.37±0.81	2.44±0.48	0.02±0.01
V209D	01.76±0.10	.0033	09.92±1.86	0.20±0.07	0.13±0.04
A211G	21.51±4.33	.0408	09.27±1.58	0.12±0.04	0.10±0.02
A302G	23.89±5.17	.0453	49.64±6.69	0.06±0.01	0.02±0.01
W377A	0.88±0.02	.0016	01.74±0.80	0.15±0.03	0.08±0.01
D382A	02.05±0.12	.0039	05.30±2.90	0.22±0.01	0.30±0.03
S404A	09.12±0.42	.0173	03.03±1.46	0.18±0.06	0.60±0.12
K487A	0.53±0.21	.0010	n.d.	n.d.	n.d.

*The kinetic parameters were determined by using non-linear fitting method (using GraphPad Prism 5, GraphPad Software, San Diego, California, USA, www.graphpad.com), n.d. – not detectable.

The enzyme activity was measured by radioactivity based assay as described in the “Materials and Methods”. The concentrations of Palmitic acid, ATP and CoenzymeA used in the reaction were 50 µM, 2 mM and 1 mM, respectively. For the measurement of K_m_, a range of substrate concentrations was used: Palimitic acid (1.5–60 µM), ATP (0.03–4 mM) and CoenzymeA (0.02–1.5 mM).

Kinetic parameters of the FadD13 mutants with respect to the native protein were analyzed to investigate the role of the targeted residues in determining the affinity of Palmitic acid, ATP and CoA. Fig. 6 and Table 2 show a comparison of K_m_ values for Palmitic acid (Fig. 6B), ATP (Fig. 6C) and CoA (Fig. 6D). The K_m_ values for the mutant K487A were not determined due to limitations in the detection of product formed as the mutant exhibited a highly reduced activity.

The K_m_ values determined for Palmitic acid (C_16_) are shown in Fig. 6B. The most drastic effect was observed in the case of mutants W377A and S404A with ∼10 times and ∼6 times reduction in their K_m_ values signifying that substitution of residues Trp^377^ and Ser^404^ to alanine results in the enhanced affinity towards palmitic acid binding. All other mutants except A302G exhibited a similar behaviour, though, to a lesser extent. The mutant A302G exhibited ∼2.5 times enhanced K_m_ value. A change in the K_m_ values of all the mutants undoubtedly points out the importance of the selected residues in the enzymatic activity. Due to a significant inhibition of the activity in the presence of α-cyclodextrin, which was essential for solubilization of C_24_/C_26_ fatty acids, the K_m_ values for these fatty acids were not determined.

In agreement with the proposed role of Lys^172^ in the ATP/AMP binding, the K172A mutant showed 10 times higher K_m_ value (1520 µM) for ATP in comparison to the native protein (Fig. 6C) suggesting that K172A is defective in the ATP binding. In contrast, the K_m_ value of CoA for K172A mutant was reduced by ∼3.5 times (Fig. 6D). The mutation of residue Ala^302^, present in the adenine binding motif, resulted in a significant decline in the K_m_ values for both ATP as well as CoA as compared to the native protein suggesting the importance of this residue in the reaction. Although, the mutants V209D and A211G exhibited altered activities, the change in activity was not accompanied by any apparent alterations in the K_m_ values for ATP and CoA in comparison to the native protein. The contribution of Ser^404^ in binding to CoA was demonstrated by a 4-fold enhancement in the K_m_ value for CoA (Fig. 6D). Residues Asp^382^ and Trp^377^ represent the signature motif of Fatty Acyl-CoA Synthetases important for their enzymatic function. The mutation of Asp^382^ was found to reduce the enzyme activity along with a marginal increase in the K_m_ values for ATP and CoA. The mutation of Trp^377^ resulted in a drastic reduction in the enzyme activity (upto 87%) suggesting its involvement in the function of enzyme, however, no significant change was observed in the K_m_ values for ATP as well as CoA (Fig. 6C, 6D).

### Evaluation of Structural Changes in FadD13 Induced by Ligand Binding and Site-Directed Mutations

The protection of FadD13 against degradation by proteinase K was investigated. The presence of both ATP and palmitic acid together resulted in a significantly enhanced protection towards proteolysis (Fig. S2A), whereas only a marginal protection was observed, when either of the two was included in the reaction indicating a conformational change induced by these ligands. The role of ATP in inducing a conformational change in FadD13 was further confirmed by a drastic reduction in the fluorescence intensity of the protein observed in the presence of ATP (Fig. S2B). The addition of CoA did not exhibit any influence on the proteolytic degradation (data not shown). Moreover, the degradation pattern of FadD13 in the absence or presence of these ligands was comparable indicating that these ligands did not alter the sites of proteolytic cleavage.

To gain an insight into the role of targeted residues in the conformational changes, the native FadD13 and the mutants were compared for their susceptibility to proteolysis. The mutants K172A, V209D and W377A exhibited a slightly increased susceptibility to proteolysis in comparison to the native protein, while the mutants A211G and A302G exhibited a reduced rate of proteolytic degradation (Fig. S2C). The mutants K487A, S404A and D382A showed no significant differences in their proteolytic susceptibility to proteinaseK in comparison to the native protein. However, the native FadD13 as well as the mutants exhibited a similar fragmentation pattern (data not shown).

### Deciphering the Role of Targeted Residues in the Secondary Structure of FadD13

**Figure 7:**
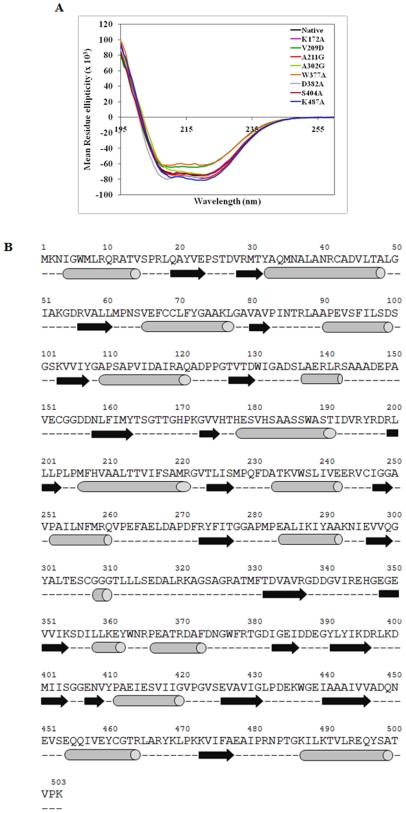
Role of targeted residues in the secondary structure of FadD13. A. Far-UV Circular Dichroism spectra of native FadD13 (NP) and its mutants. An average of three scans was recorded for each sample and the spectrum for each protein was measured in duplicate samples (mean spectrum is depicted here). Spectra were recorded at a protein concentration of 0.2 mg/ml in 10 mM sodium phosphate buffer, pH 8.0. The data were converted to molar ellipticity units. B. Secondary structure prediction of FadD13. The prediction of secondary structure was carried out by using PSIPRED and JPred softwares. Cylinders, arrows and dash represent α-helix, β-sheet and coils, respectively.

To assess the secondary structural features of FadD13, Far-UV circular dichroism spectroscopy was performed (Fig. 7A). The spectrum of native FadD13 indicated it to be a highly structured protein with characteristic double minima at 210 nm and 222 nm and it belonged to α/β class of proteins comprising of 34% α helix, 16% β sheet, 19% turn and 31% random coil (Table 3). This content is in close agreement with those predicted by PSIPRED [29], [30] and Jpred [31] based on sequence analysis (Fig. 7B) with a secondary structure content of 36% α helix, 21% β sheet, and 44% coiled conformation. Most of the mutants showed a similar secondary structure content as that of the native protein except for the mutants V209D and W377A (Table 3), which displayed altered spectra attributable possibly to an increased β sheet content (33% and 31%, respectively).

**Table 3:** Secondary structure content of native FadD13 and its mutants.

Secondary structure	Native FadD13	K172A	V209D	A211G	A302G	W377A	D382A	S404A	K487A
α-helix	34.3%	33.0%	27.9%	37.0%	39.9%	35.7%	28.4%	33.7%	35.9%
β-sheet	15.9%	19.8%	32.9%	13.3%	07.2%	31.3%	26.4%	16.7%	14.1%
Turn	18.9%	16.4%	10.1%	18.7%	22.2%	06.9%	13.6%	16.8%	18.8%
Random	30.8%	30.8%	29.0%	30.9%	31.3%	26.1%	31.6%	32.8%	31.3%

### Intrinsic Fluorescence and Thermal Stability of the Native FadD13 and Its Mutants

**Figure 8:**
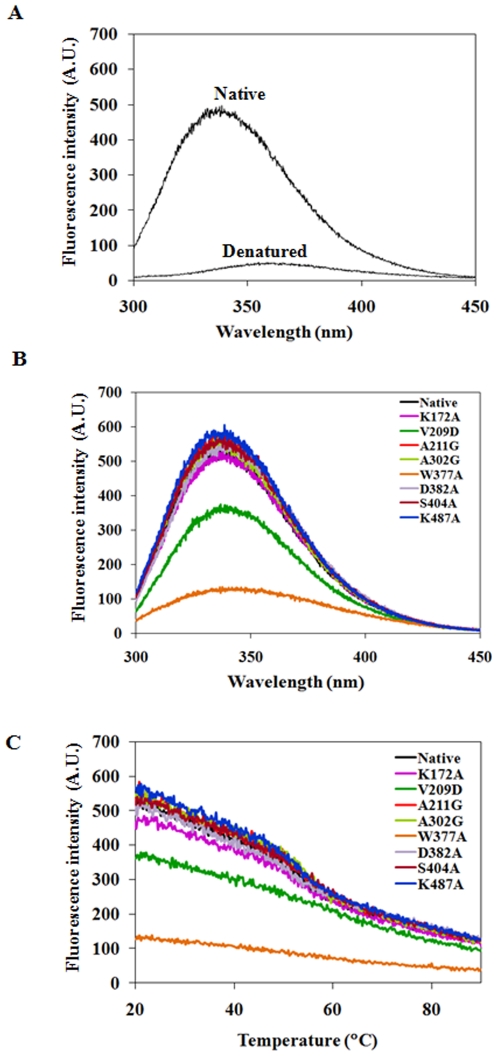
Measurement of intrinsic fluorescence of FadD13 and its mutants. A. Effect of chemical denaturation on fluorescence emission spectrum of native FadD13. The protein was incubated with 7.2 M guanidine hydrochloride for 24 hours. The figure shows the fluorescence spectra for native and denatured protein (excitation wavelength - 280 nm). B. Fluorescence emission spectra of native FadD13 (NP) and its mutants (excitation wavelength - 280 nm). C. Fluorescence emission spectra of native FadD13 (NP) and its mutants as a function of temperature. A protein concentration of 0.1 mg/ml in 50 mM sodium phosphate buffer pH 8.0 was used for the measurements. The Excitation and emission wavelengths used were 280 nm and 340 nm, respectively.

Fluorescence spectroscopy was employed to ascertain subtle structural differences between the native FadD13 and its mutants. FadD13 contains 16 tyrosine and 7 tryptophan residues distributed all over the protein sequence. The fluorescence spectrum of the native protein exhibited an emission maximum at a wavelength of 340 nm after excitation at 280 nm, while a red shift of 22 nm along with a substantially reduced intensity was observed in the case of 7.2 M guanidine hydrochloride denatured protein (Fig. 8A) indicating that the tryptophan and tyrosine residues that were buried in a non-polar environment in the native state get exposed to polar solvent upon unfolding. For the mutants V209D and W377A, the fluorescence intensity was drastically reduced in comparison to the native FadD13 showing a highly perturbed tertiary structure (Fig. 8B). The other mutants exhibited spectra comparable to that of the native protein (Fig. 8B).

Thermal denaturation studies were performed to compare the stability of the native FadD13 and its mutants (Fig. 8C). The native protein as well as most of the mutants showed an apparent melting temperature (T_m_) of 54°C and exhibited a slow cooperative behaviour in the opening up of the structure. However, the absence of a distinct thermal transition along with a non-cooperative behaviour of unfolding was observed in the cases of V209D and W377A further confirming the loss of tertiary structure in these mutants.

### Influence of Site Directed Mutations on the Aggregation Properties of FadD13

**Figure 9:**
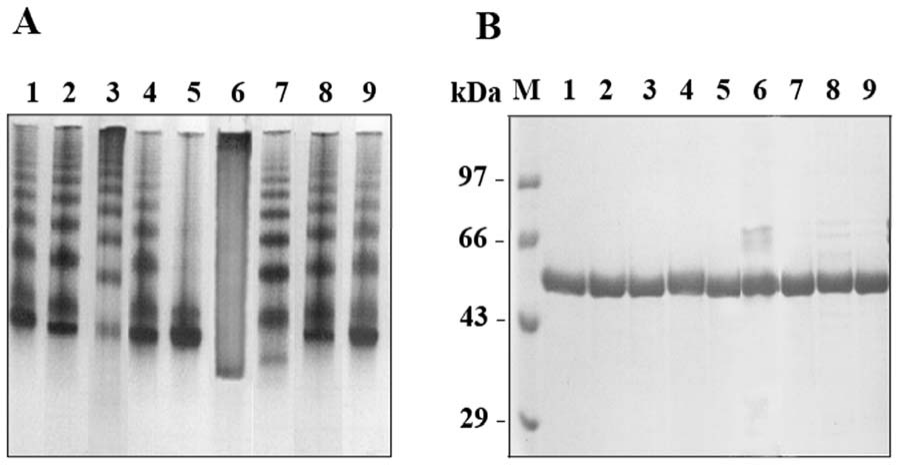
Aggregation properties of native FadD13 and its mutants. Analysis of native FadD13 and its mutants on 7.5% non-reducing non-denaturing polyacrylamide gel (A) and 10% SDS polyacrylamide gel (B). M-molecular weight markers. For both A and B - lane 1- native FadD13, lane 2- K172A, lane 3- V209D, lane 4- A211G, lane 5-A302G, lane 6- W377A, lane 7- D382A, lane 8- S404A and lane 9- K487A.

As seen in Fig. 9A, the mutant A302G (lane 5) completely abrogated the aggregation propensity of FadD13, as analyzed by native polyacrylamide electrophoresis, resulting in a single major band corresponding to dimeric species as opposed to the presence of aggregates in addition to the predominance of dimeric species in the case of native FadD13 (lane 1). This observation suggests that the presence of a glycine residue at this position influences protein's conformation, which helps in preventing the formation of non-specific disulfide linkages, the major cause of aggregation. Mutation of the residues Ala^211^ and Lys^487^ (lane 4 and 9) did not influence the aggregation of protein to any appreciable extent. However, a marginally higher aggregation was observed, when the residues Lys^172^ (lane 2) and Ser^404^ (lane 8) were mutated. While the mutation of Val^209^ (lane 3) resulted in a markedly increased aggregation, the mutation of Asp^382^ (lane 7) in addition to a markedly increased aggregation also resulted in the presence of a monomeric form not seen in the case of native protein implying the role of this residue in dimerization. Mutation of Trp^377^ resulted in the highest aggregation observed (lane 6), some of which could not be dissociated under denaturing conditions (Fig. 9B, lane 6). The purified protein, if instantly analyzed on SDS-PAG, exhibited high purity with a single band, however, the aggregation started soon thereafter and even the stability of the protein seemed compromised as aggregated versions along with the degradation product became more pronounced as time proceeded.

### Three Dimensional Structure Prediction of FadD13 by Using Homology Modeling

**Figure 10:**
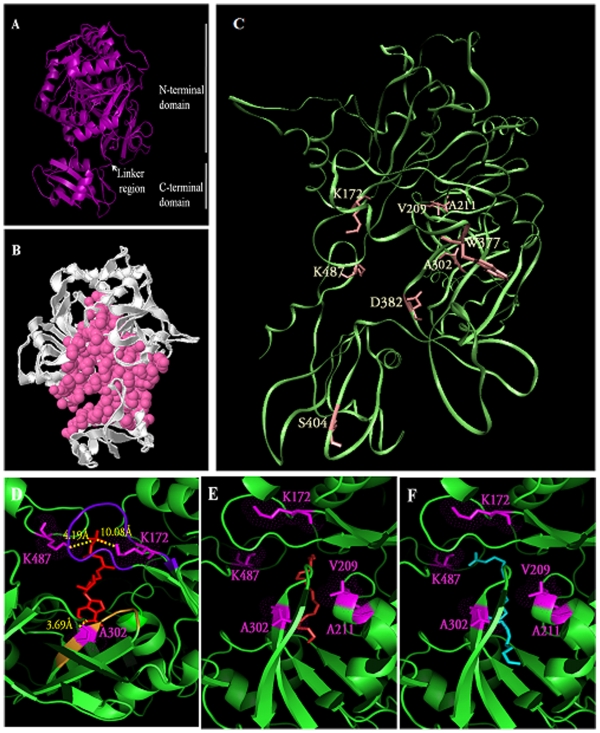
Homology modeling based three-dimensional structure of FadD13. A. Overall structure of FadD13 model. The FadD13 model was generated by using the SWISS-MODEL server based on the template with pdb id: 2QVY. A cartoon representation of FadD13 structure shows the major domains and the connecting linker region. The figure was prepared by using the PyMol Molecular Viewer. B. Active site of FadD13. The figure depicts the predicted active site (shown as pink balls). The active site prediction as well as the preparation of the figure was carried out by using the CastP server. C. Location of the residues selected for mutagenesis. The 8 residues targeted for mutation in this study are shown in stick model (pink). The figure was prepared by using VMD 1.8.06. D. Docking of ATP to the active site. The figure shows the interaction of ATP (red) with the residues (magenta, in stick model) that were found to be critical for ATP binding by mutational studies. The phosphate binding loop (motif I) and the adenine binding motif (motif II) are shown in blue and orange, respectively. The interaction between the residues and ATP are shown in yellow with the inter-atomic distances marked (also in yellow). E. Docking of Palmitic acid (C_16_) to the active site. F. Docking of Tetracosanoic acid (C_24_) to the active site. Figure E and F shows Palmitic acid (red) and Tetracosanoic acid (cyan), respectively, at the active site of FadD13 in close proximity to the critical residues (magenta) identified on the basis of mutational analysis. Figures D, E and F were prepared by using PyMol Molecular Viewer.

While the efforts to crystallize FadD13 and determination of its structure are underway in our laboratory, we performed molecular modeling of FadD13 to validate our mutational studies as well as the substrate preference of FadD13. The best model was generated based on the template 4-Chlorobenzoyl-CoA Ligase/Synthetase of *Alcaligenes sp*. al3007 [32], having a sequence identity of 30% and a homology of 50% with FadD13. The general fold of the modeled structure of FadD13 (Fig. 10A) resembles those of the known crystal structures of homologous proteins belonging to the Fatty Acyl-CoA Synthetase family [23], [28], [33]. The overall structure is composed of a large N terminal domain (residues 1–397) and a small C terminal domain (residues 402–498) that are connected with a linker region.

### Prediction of the Substrate Binding Site and Docking of Ligands

The substrate binding site was predicted by using the CastP server [34]. Although, the server predicted a number of active sites, only the first hit having the largest area seemed to be correct based on the crystal structures of other homologous proteins (Fig. 10B). The predicted active site covered parts of both the N and C terminal domains along with the cleft region placed between both the domains. Moreover, the active site was similar to that seen in other homologous proteins [23], [33]. It can also be observed that most of the residues targeted for the mutational studies resided within the predicted active site (Fig. 10C) thus justifying the influence of the mutation of these residues on the enzyme's function as indicated by the biochemical studies. The docking of ATP and Palmitic acid (C_16_) at the active site verified the modeled structure and corresponded well with the results of the mutational studies. Fig. 10D demonstrated the molecular interaction of ATP with residue Ala^302^ positioned at ∼3.7 Å from the adenine moiety of ATP. The cognate glycine residue has been shown to interact in other homologs similarly [23]. Fig. 10D also depicted weak electrostatic attraction between the phosphate backbone of ATP molecule and the nearby Lys^172^ and Lys^487^ residues. An orchestration of the residues, shown to play a critical role in protein function, in the vicinity of Palmitic acid molecule (Fig. 10E) strongly emphasizes the importance of these residues in the enzyme activity. Fig. 10F shows the docking of Tetracosanoic acid (C_24_) at the active site. A higher binding energy of docking (data not shown) in the case of C_24_ fatty acid as compared to C_16_ fatty acid supports the preference of the protein towards higher chain length fatty acids observed in activity analysis.

## Discussion

*mymA* is an important operon of *M.tb* due to its involvement in the remodeling of the cellular envelope under stressful conditions and in the intracellular survival of the pathogen [8], [9], [10]. It is comprised of seven genes and the last gene encodes a Fatty Acyl-CoA Synthetase (Rv3089, *fadD13*). Due to essential role of *mymA* operon in the pathogenesis of *M.tb*, FadD13 represents an important drug target. A few other mycobacterial Acyl-CoA Synthetases namely MenE (involved in menaquinone biosynthesis) and FAAL28 and FAAL19 (involved in Fatty acid metabolism) have also been targeted for the development of antitubercular drugs and small molecule inhibitors against these enzymes have been developed [15], [16]. Besides, Fatty Acyl-CoA Synthetase inhibitor Triacsin C has been widely studied as a therapeutic agent for the treatment of artherosclerosis and certain kind of tumors [35], [36].

In the present study, we have characterized the important domains of FadD13 by mutating residues belonging to these regions and identified various residues important for the enzymatic activity, structural integrity and substrate binding. An extremely compromised activity of K487A mutant along with a tremendously low K_cat_ value (Table 2) suggests a crucial requirement of Lys^487^ for the enzymatic activity, which agrees well with the role of the cognate residues in other homologous proteins [24], [25]. The structure of FadD13 shows a close proximity of the side chain of Lys^487^ with the phosphate group of ATP (Fig. 10D) and therefore, is an essential residue for adenylate formation. Hence, the loop region harboring this residue, owing to its implication in the mechanism of enzyme action could provide an important target for the design of inhibitors against FadD13. Moreover, all other parameters measured in this study such as the aggregation property, secondary structure content, susceptibility to proteolysis and fluorescence spectra depicted comparable results in the case of this mutant and the native protein, suggesting thereby that the marked reduction in the activity may primarily signify the contribution of the target residue in the enzyme catalysis and not an influence on the structure and stability of the protein.

Intrinsic fluorescence of proteins is considered to be an important measure of their three-dimensional structure and is sensitive to subtle changes in the local environment of the tryptophan/tyrosine residues [37]. The mutants V209D and W377A exhibited a remarkable reduction in their intrinsic fluorescence (Fig. 8B) implying thereby perturbations in their tertiary structures along with conformational changes, which are indicative of significant differences in the environment of tryptophan/tyrosine residues in these two mutants as compared to the native protein. It is noteworthy that FadD13 has 7 tryptophan and 16 tyrosine residues; however, mutation of a single tryptophan (Trp^377^) resulted in almost 80% loss of fluorescence, indicating a highly perturbed structure of this mutant as compared to the native protein (Fig. 8B), which is also substantiated by the absence of a distinct transition in its thermal denaturation pattern (Fig. 8C). It was observed that heat denaturation resulted in a significant reduction in the fluorescence intensity of the native FadD13 implying the exposure of tryptophan/tyrosine residues to the polar environment due to unfolding leading to the quenching of fluorescence. However, heat denaturation had only a marginal effect on the fluorescence intensity of the mutant W377A (data not shown), thus pointing out that either the tryptophan/tyrosine residues were already exposed to the polar environment or the local environment of these residues differs in the case of this mutant as compared to the native protein. A higher susceptibility exhibited by these mutants towards proteinase K (Fig. S2C) suggests loosening of their tertiary structure and a greater amenability to protease action. Moreover, an exceptionally high aggregation observed in these cases (Fig. 9A) is consistent with increased β-sheet content (Table 3) as conversion of α helices into β sheets often underlines the process of protein aggregation [38]. As demonstrated by localization studies, the mutation of Trp^377^ to alanine resulted in the formation of inclusion bodies (Fig. S1); in contrast to the native FadD13, wherein about 80% of the synthesized protein localized in the soluble portion, the majority of the mutant protein was localized in the insoluble portion suggesting the involvement of residue Trp^377^ in the proper folding of the native FadD13. Besides, two other mutants namely V209D and D382A also caused increased formation of inclusion bodies and aggregation of protein. However, the extent of influence on the folding of protein was much lesser in these cases in comparison to the W377A mutant; in case of the V209D and D382A mutants, the synthesized protein was localized equally in the soluble and insoluble portions (Fig. S1). An interesting corollary to these results pertains to the presence of a small amount of monomeric form of FadD13 in the case of mutant D382A as revealed by gel filtration studies and native PAGE (Fig. 9A), which was not observed in the case of any other mutant or native FadD13. These observations suggest the possibility that Asp^382^ may help in optimizing the interactions required for the dimerization of this protein. Taken together, fluorescence studies, proteinase K proteolysis, native PAGE analysis and localization studies strongly suggest the involvement of residues Trp^377^, Val^209^ and Asp^382^ in maintaining the structural integrity of FadD13.

Studies on the catalytic activity and substrate affinity of FadD13 provided interesting insight into the role of selected residues in the enzyme catalysis. A significantly diminished activity exhibited by the mutants W377A, V209D and D382A (Fig. 6A) is in agreement with the marked influence of the mutations on the structural integrity of the protein as described in the above section. The mutation of Ser^404^ to alanine resulted in a 40% reduced activity along with a concomitant 4-fold increment in the K_m_ value for CoA (Fig. 6) implicating this residue in the binding of CoA. This observation is in agreement with the crystal structure of Acteyl-CoA Synthetase from *Salmonella enterica*, which showed the interaction of a Serine residue at a similar position with the pantothenate group of CoA [28]. According to the FadD13 structure, residue Ser^404^ lies at the far end of the active site (Fig. 10C), however, the conformational change brought about in the structure as a result of ATP binding (as discussed below) thus resulting in bringing this residue closer to the active site cannot be ruled out. Similarly, the substitution of Lys^172^ to alanine marked the importance of this residue in the ATP binding as was evident from ∼60% loss of the enzymatic activity along with a concomitant increase in the K_m_ value for ATP (Fig. 6). The corresponding lysine residues in several Fatty Acyl-CoA Synthetases have been implicated in the binding to the phosphate group of ATP via electrostatic interactions [21]. Interestingly, a simultaneous 6-fold decrease in the K_m_ value for CoA was also observed, pointing out that the mutation of Lys^172^ to alanine results in a higher affinity for CoA binding. However, the increased CoA affinity appears to be insufficient to compensate for the defect in the ATP binding. The mutation of Ala^302^ to glycine resulted in a significantly higher activity and ∼3.7 fold reduction in the K_m_ value for ATP (Fig. 6) indicating that the presence of a glycine residue facilitates the binding of ATP. It has been earlier reported that the cognate glycine residue has been involved in adenylate binding [23]. While these observations show the importance of this locus in the enzyme activity, we noted that alanine at this position features only in a few mycobacterial Fatty Acyl-CoA Synthetases, whereas most other FadD enzymes from mycobacteria as well as from other origins have the prevalence of a glycine residue at this locus. As observed in the structure (Fig. 10D), the ATP binding site mainly comprises of two loops encompassing motif I and motif II. A drastic effect on the K_m_ values of ATP for K172A and A302G mutants was confirmed by the interaction of these residues with the ATP molecule.

FadD13 showed a markedly reduced proteolysis when the protein was pre-incubated with ATP (Fig. S2A), which suggests that adenylate formation results in the conformational changes in the structure of FadD13 leading to more stability and resistance to the action of proteinase K. A concomitantly reduced fluorescence intensity and aggregation property of FadD13 in the presence of ATP (Fig. S2B) further confirms the positive influence of this ligand binding on the structural integrity of the protein. The structural studies with ttLC-FACS have revealed that the presence of ATP renders the protein in a more stable and compact form [23]. This kind of compactness in the structure of FadD13 will lead to reduced inter-atomic distances between phosphate group of ATP and the P-loop of the protein thus making the interactions for binding more stronger. In the present study, although, both fatty acid as well as ATP binding protected FadD13 from proteolysis, this protective effect was markedly enhanced when the protein was incubated with both the ligands together (Fig. S2A) and the effect was more than additive suggesting that a simultaneous binding of these ligands may have a positively synergistic effect on the conformation and rigidity of the protein.

The preference of FadD13 for fatty acids of various chain lengths was explored. Although, the protein utilized a number of fatty acids as substrate such as propionic acid, hexanoic acid, decanoic acid, lauric acid, palmitic acid, stearic acid and oleic acid (data not shown) as is the case with many other known Fatty Acyl-CoA Synthetases [22], [39], it exhibited a significantly higher activity for C_26_ and C_24_ fatty acids as compared to C_16_ fatty acids (Fig. 3). Moreover, the docking studies corroborate with the activity data showing a higher binding energy for C_24_ fatty acid as compared to C_16_ fatty acid (Fig. 10E and 10F). In view of our earlier observations, the preference of FadD13 for C_26_/C_24_ fatty acids further confirms the role of *mymA* operon in remodeling the envelope of intracellular *M.tb* under acidic conditions [9]. Fisher et. al. have demonstrated that under acidic conditions, a 4–5 fold upregulation of the *mymA* operon is accompanied by a two fold reduction in the expression of FAS-II operon associated genes [7] responsible for the synthesis of meromycolates [40], which on conjugation with very long chain fatty acids like C_24_ and C_26_ lead to the formation of mycolic acids [40]. Thus, downregulation of FAS-II would lead to an accumulation of these long chain fatty acids, which are known to be toxic for the cell [41]. Therefore, it is believed that a concomitant upregulation of the *mymA* operon under these conditions will result in the utilization of these very long chain fatty acids, effectively modifying and further transferring them to appropriate biological acceptor on the cell wall for its remodeling [9]. Thus, upregulation of the *mymA* operon might serve a two fold purpose, i.e., it can serve as a sink for the accumulated C_24_ and C_26_ fatty acids to prevent toxicity as well as generate appropriate lipids required by the pathogen under the acidic conditions. However, the exact product of *mymA* operon needs to be elucidated.

FadD13 depicted biphasic kinetics for fatty acids (Fig. 2A, 2B) in contrast to the classical Michaelis-Menten kinetics reported in the case of other analogous proteins [42], [43]. The precise reason for this observed biphasic kinetics for fatty acid cannot be discerned at present, although, several plausible reasons for such kinetic behavior could involve the presence of multiple substrate binding sites with different binding affinities or binding of substrate in more than one orientation with different affinities [44], [45]. The understanding of this rather unusual kinetic behavior would require further investigation.

In conclusion, our study provides a significant understanding of the FadD13 protein including the identification of residues important for its activity as well as in the maintenance of structural integrity. Our results demonstrate Lys^487^ as an essential residue in the activity of FadD13 whereas Trp^377^ and Val^209^ were established to be important residues in the structural stability. Comparison of the kinetic parameters related to FadD13 and its mutants showed residues Lys^172^ and Ala^302^ to be involved in the binding of ATP to the active site and Ser^404^ in the binding of CoA. We also show a synergistic influence of fatty acid and ATP binding on the conformation and rigidity of FadD13. Additionally, the docking studies using the homology model of FadD13 substantiate the observations of mutational studies. FadD13 exhibits preference for C_26_/C_24_ fatty acids, which in the light of earlier reported observations further confirms the role of *mymA* operon in remodeling the cell envelope of intracellular *M.tb* under acidic conditions. We believe that the findings of this study would provide valuable inputs in the development of inhibitors against *mymA* operon, an important target for the development of antitubercular drugs.

## Materials and Methods

### Materials, Bacterial Strains and Growth Conditions

Molecular biology methods employed in this study were performed according to the standard protocols by Sambrook and Russell [46]. All radiolabeled chemicals were procured from American Radiolabeled Chemicals, Inc. (St. Louis, MO, USA) and other reagents were obtained from Sigma-Aldrich Inc. (St. Louis, MO, USA). Quik change II XL site directed mutagenesis kit was obtained from Stratagene (La Jolla, CA, USA). Ni-NTA superflow resin was procured from Qiagen (Spoorstraat, KJ Venlo, The Netherlands). PAGE purified mutagenic primers were obtained from Sigma-Aldrich Inc. (St. Louis, MO, USA). Gel Filtration standard markers were obtained from Biorad Laboratories (Hercules, CA, USA). *E.coli* BL21 (λDE3) cells were grown in Luria Bertani (LB) broth at 37°C with constant shaking at 200 rpm. *Mycobacterium smegmatis*(*M.sm*) mc^2^155 cells were grown by using either Difco Middlebrook (MB) 7H9 supplemented with 0.5% glycerol, 0.2% tween-80 at 37°C with constant shaking at 200 rpm or on Difco Middlebrook 7H11 agar. The Difco Middlebrook media were obtained from Becton Dickinson and Company (Sparks, MD, USA). Whenever appropriate, antibiotics were added at a concentration of 50 µg/ml ampicillin (Amp), 25 µg/ml Kanamycin (Kan) and 150 µg/ml hygromycin (Hyg) for *E.coli*. For *M.sm*, hygromycin and Trimethoprim (Trim) were used at a concentration of 50 µg/ml.

### Cloning of *fadD13* Gene

Based on the sequence available from the EMBL/Genbank, the primers 5′catatgaagaacattggctggatgctcag 3′ (forward primer containing *Nde*I restriction site) and 5′ctcgagtcacttcggcaccgtcgccg 3′ (reverse primer containing *Xho*I restriction site) were used to amplify the gene encoding Fatty Acyl-CoA Synthetase (Rv3089, FadD13) by using *M.tb* genomic DNA as template. The PCR amplicon was cloned into plitmus38 at *Eco*RV site resulting in plit38.fad. For expression studies, the gene was excised out by using *Nde*I and *Xho*I restriction enzymes and was cloned into pET28c at the same sites resulting in pET28c.fad.

### Expression and Purification of Recombinant FadD13

*E.coli* BL21 (λDE3) cells were transformed with pET28c.fad, the transformants were grown to mid-logarithmic phase in LB media containing 25 µg/ml of kanamycin and synthesis of FadD13 protein was induced by the addition of 1 mM isoproryl-1-thio-β-D-galactopyranoside (IPTG) and the cells were harvested after incubation at 30°C for 3 hours with a constant shaking at 200 rpm. Induction in the case of mutant W377A was carried out at 18°C for 16 hours. The harvested cells were suspended in lysis buffer (50 mM NaH_2_PO_4_, 300 mM NaCl, 10 mM imidazole, 20% glycerol, 1 mM PMSF, 2 mM β-mercaptoethanol, pH 8.0) and lysed by using French press (SLM Instruments, Inc., Urbana, IL, USA). Purification was carried out by Ni-NTA agarose affinity chromatography. Briefly, the cell-extract-Ni-NTA agarose slurry was kept for binding on a rotary shaker for 2 hours at 4°C. After removal of unbound proteins (at 2000 g for 2 minutes), the resin was washed twice with lysis buffer. For higher stringency, washings were repeated twice with lysis buffer containing 20 mM imidazole and once with lysis buffer containing 50 mM imidazole. The protein was eluted by using lysis buffer containing 250 mM imidazole and purification was monitored on 10% SDS-polyacrylamide gel. The purified his-tagged protein was dialyzed against 1X PBS before use. Protein concentration was determined by Bradford's method [47] with bovine serum albumin as the standard.

### Determination of Oligomeric Nature of FadD13

The oligomeric status of FadD13 was determined by using Mycobacterial-Protein Fragment Complementation [20]. For this, the gene encoding FadD13 was excised out from pET28c.fad, end repaired by Klenow polymerase and cloned into pUAB300 and pUAB400 (digested with *Bam*HI and *Hin*dIII, respectively) resulting in pUAB300.fad and pUAB400.fad leading to production of FadD13_[F1,2]_ and FadD13_[F3]_, respectively. The digested vectors were end repaired and dephosphorylated before cloning. *M.sm* mc^2^155 cells were independently electroporated with M-PFC plasmids producing either (1)–FadD13_[F1,2]_/FadD13_[F3]_, (2)–*hsp60*_[F1,2]_/*hsp60*_[F3]_, (3)–GCN4_[F1,2]_/GCN4_[F3]_, (4) –FadD13_[F1,2]_/*hsp60*_[F3]_, (5)–*hsp60*_[F1,2]_/FadD13_[F3]_ and the transformants were selected on MB 7H11 agar containing 25 µg/ml kanamycin and 50 µg/ml hygromycin. The transformants were analyzed for oligomerization of FadD13 by their ability to grow in the presence of 50 µg/ml Trimethoprim [20].

### Determination of Subunit Assembly by Gel Filtration Chromatography

A sephadex G-200 column (2.5 cm×92 cm) equilibrated with 20 mM Tris-HCl pH 8.0, 0.1 M NaCl, 10% glycerol and 0.02% sodium azide was used to determine the subunit assembly of FadD13. 5 mg of purified FadD13 was resolved on the column and its molecular weight was determined by comparison with the known molecular weight standards.

### Measurement of Activity of Fatty Acyl-CoA Synthetase

The Fatty Acyl-CoA Synthetase activity was measured by using the enzymatic assay as described earlier [17]. The purified protein was incubated with 10 mM DTT for 2 hours prior to its use to ensure the removal of any intermolecular disulfides leading to formation of aggregates. Briefly, the reaction mixture contained 2 mM ATP, 10 mM MgCl_2_, 1 mM CoA, 50 µM ^14^C radiolabeled palmitic acid and 1 µg protein. The reaction volume was made up to 15 µl by 20 mM Tris-HCl pH 8.0. The reaction was carried out at 37°C for 5 minutes and was terminated by the addition of 5 µl of 10% acetic acid. The samples were resolved on silica coated TLC plates at 4°C by using the solvent system Butanol∶water∶acetic acid (80∶40∶25). The resolved radioactive bands were visualized by using phosphorimager (model-FLA-9000, FUJIFILM Corporation, Minato-ku, Tokyo, Japan) and the band intensity was quantified by using the Multi Gauge software (FUJIFILM Corporation, Minato-ku, Tokyo, Japan). The amount of radiolabeled product formed was calculated on the basis of comparison with the known amounts of radiolabeled fatty acids.

### Preference of FadD13 for Fatty Acid Chain Length

^14^C radiolabeled palmitic acid (C_16_), tetracosanoic acid (C_24_) and hexacosanoic acid (C_26_) were used as substrates for FadD13. Due to the insoluble nature of C_24_ and C_26_ fatty acids, they were dried to completely remove alcohol and resuspended for solubilization in 10 mg/ml of α-cyclodextrin prepared in 20 mM Tris-HCl pH 8.0 [18], [19] to a final concentration of 34.1 µM and 48.5 µM, respectively. The activity of FadD13 was determined by using 15 µM of each of the fatty acids separately in the presence of equal percentage of α-cyclodextrin.

### Site Directed Mutagenesis of FadD13

Site directed mutagenesis was carried out by using Quik change II XL site directed mutagenesis kit and mutagenic primers. Sequences of the primers used are given in Table 1. The plasmid plit38.fad (4.3 Kb) containing the gene encoding FadD13 was used as the template for mutagenesis. Mutagenesis was carried out as per the manufacturer's recommendations and the resulting mutations were confirmed by nucleotide sequencing. For expression studies, the mutated insert was excised out of plit38.fad and cloned into pET28c by using the same cloning strategy as described above. The expression and purification procedure was performed similarly as for the native protein.

### Limited Proteolysis

Limited proteolysis was performed by incubating 15 µg of protein and proteinase K in a ratio of 1∶1000 or 1∶2000 (protease∶protein by mass) in 1X PBS pH 7.2 containing 10 mM MgCl_2_ at 25°C. Ligands, when present, were added prior to the addition of protease at a concentration of 2 mM (ATP), 1 mM (CoA) and 50 µM (palmitic acid). Samples were withdrawn at various time intervals and immediately boiled at 100°C in the presence of SDS gel loading dye. The pattern of proteolysis was monitored by electrophoresis using 10% SDS-polyacrylamide gel.

### Circular Dichroism Studies

Far-UV CD spectra were recorded on J-815 spectropolarimeter (JASCO Corporation, Hachioji-shi, Tokyo, Japan). An average of 3 scans was taken and the data was converted to molar ellipticity units by using the formula [θ]  =  millidegrees/(pathlength in millimeters × molar concentration of protein × number of residues). The spectra were obtained at an interval of 0.1 nm with a scanning speed of 50 nm/min at 20°C by using a 0.1 cm path length quartz cuvette. A protein concentration of 0.2 mg/ml in 10 mM sodium phosphate, pH 8.0 was employed.

### Fluorescence Studies

All the fluorescence measurements were carried out on a Cary Varian Eclipse Fluorescence spectrophotometer (Varian, Inc. Hansen Way, Palo Alto, CA, USA) having an attached Peltier temperature controller. A protein concentration of 0.1 mg/ml in 50 mM sodium phosphate, pH 8.0 was used for the measurements. An excitation wavelength of 280 nm with an excitation slit of 2.5 nm and an emission slit of 5 nm was used and the fluorescence was recorded from 300 nm to 450 nm. Thermal denaturation experiments were performed by using a temperature range of 20°C to 90°C at an excitation wavelength of 280 nm and an emission wavelength of 340 nm at a scan rate of 1°C/min.

### Multiple Sequence Alignment, Homology Modeling and Ligand Docking of FadD13

The multiple sequence alignment of FadD13 with homologous proteins of Fatty Acyl-CoA Synthetase family was generated by using the ClustalW software [48]. The three dimensional homology model of FadD13 was generated with the SWISS-MODEL server [49], [50] by using the X-ray crystal structure of 4-Chlorobenzoyl-CoA Ligase/Synthetase of *Alcaligenes sp*. al3007 (Protein Data Base Code-2QVY) [32]. The model generated was used for subsequent studies after verification with RAMPAGE [51] and ERRAT [52] and energy evaluation by using the Swis-Pdb Viewer [53]. The active site prediction for the model was performed by using the CastP [34] server and the prediction having the largest area was selected as the best active site. The structures for ATP and Palmitic acid were obtained from the crystal structure of Human Cobalamine adenosyltransferase bound to ATP (pdb id: 2IDX) [54] and the Molecule Database [55], respectively. Structure of Tetracosanoic acid was generated by using the PRODRG software [56]. The docking studies were performed by using the software AutoDock Tools 4.1 [57] and the grid site for docking of ligands was based on the active site prediction. Ten conformations for each substrate were obtained. The best conformations were selected on the basis of combination of binding energies as well as location of the docking. Images for the FadD13 model and the docking studies were prepared by using Pymol [58] whereas the images showing the active site and location of residues selected for mutagenesis were generated by using CastP [34] and VMD [59], respectively.

## Supporting Information

Figure S1 Sub-cellular localization of FadD13 mutants. The induced culture was harvested followed by sonication of the resuspended cells. After centrifugation of the sonicated extract at 16,000 g for 30 minutes, to separate the cytosolic proteins and inclusion bodies, the localization of the native FadD13 (NP) and its mutants was studied by analyzing the fractions on a 10% SDS-polyacryalmide gel. M-Molecular weight markers, P- proteins in the inclusion bodies, S- proteins in the cytosolic fraction.
(6.15 MB TIF)

Figure S2 Study of the role of the targeted residues in the structural stablity of FadD13. A. Limited proteolysis of FadD13 in the absence and presence of substrates. A ratio of 1∶1000 and 1∶2000 (proteinase K: protein) was employed and the reactions were carried out for 10 minutes and 30 minutes by using 15Î¼g of protein. B. Fluorescence emission spectrum of native FadD13 in the absence and presence of 2 mM ATP (excitation wavelength - 280 nm). C. Limited proteolysis of native FadD13 (NP) and its mutants. The proteolysis was carried out at a proteinase K: protein ratio of 1∶2000 by using 15Î¼g of protein.
(7.83 MB TIF)
