# Supplementary figures and images for: Dissecting the Role of Critical Residues and Substrate Preference of a Fatty Acyl-CoA Synthetase (FadD13) of Mycobacterium tuberculosis
Source: PLoS One. 2009 Dec 21;4(12):e8387. doi: 10.1371/journal.pone.0008387 (PMC2793005; doi:10.1371/journal.pone.0008387)

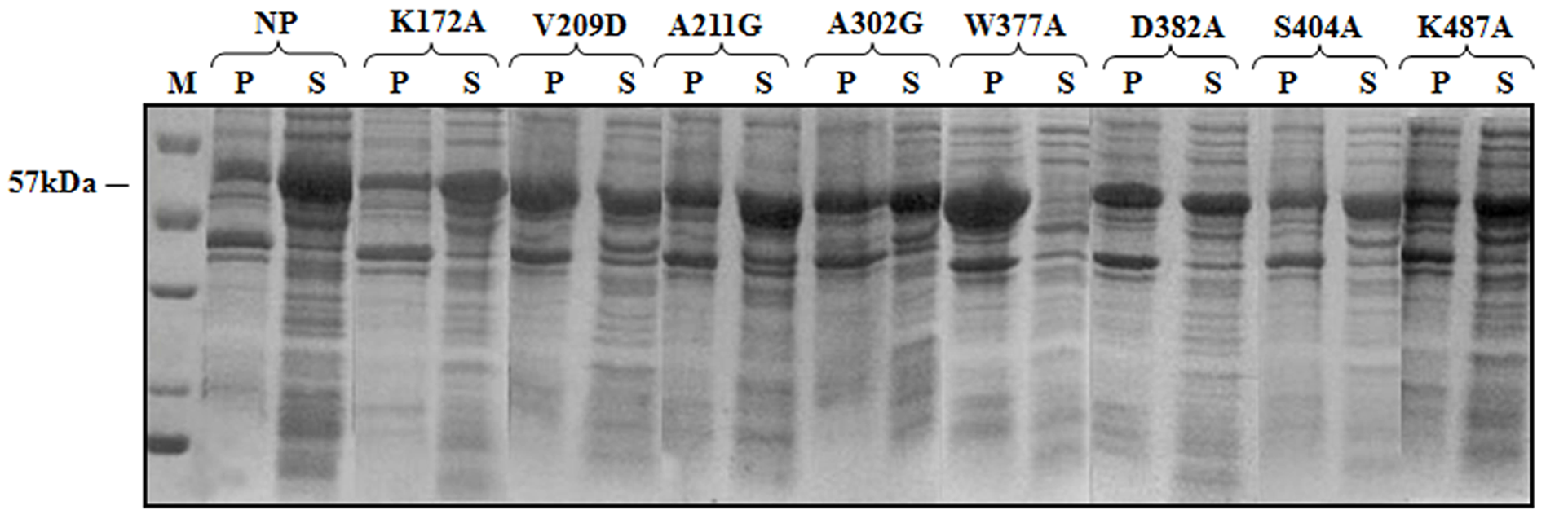

Supplement: Figure S1 — Sub-cellular localization of FadD13 mutants. The induced culture was harvested followed by sonication of the resuspended cells. After centrifugation of the sonicated extract at 16,000 g for 30 minutes, to separate the cytosolic proteins and inclusion bodies, the localization of the native FadD13 (NP) and its mutants was studied by analyzing the fractions on a 10% SDS-polyacryalmide gel. M-Molecular weight markers, P- proteins in the inclusion bodies, S- proteins in the cytosolic fraction. (6.15 MB TIF) [file pone.0008387.s001.tif]

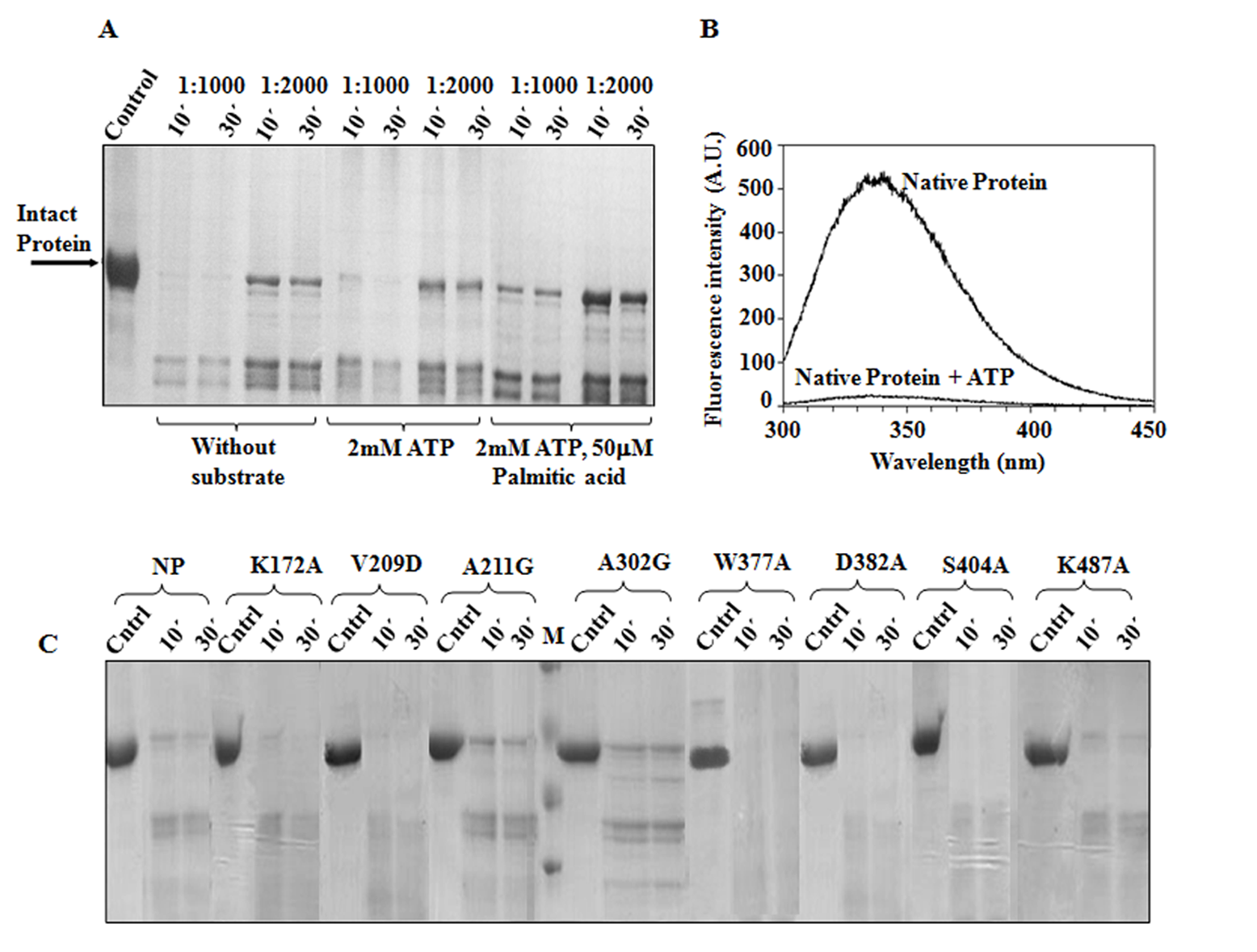

Supplement: Figure S2 — Study of the role of the targeted residues in the structural stablity of FadD13. A. Limited proteolysis of FadD13 in the absence and presence of substrates. A ratio of 1∶1000 and 1∶2000 (proteinase K: protein) was employed and the reactions were carried out for 10 minutes and 30 minutes by using 15Î¼g of protein. B. Fluorescence emission spectrum of native FadD13 in the absence and presence of 2 mM ATP (excitation wavelength - 280 nm). C. Limited proteolysis of native FadD13 (NP) and its mutants. The proteolysis was carried out at a proteinase K: protein ratio of 1∶2000 by using 15Î¼g of protein. (7.83 MB TIF) [file pone.0008387.s002.tif]
